# Hyperbaric oxygen-assisted neurological recovery in a patient with classic heat stroke: a case report

**DOI:** 10.3389/fmed.2026.1726603

**Published:** 2026-02-19

**Authors:** Xin-xin Zheng, Liu-xu Chen, Ren Cheng-Han Fan, Zhi-qiang Zhang, Sheng Chen, Xiao-yang Wang, Guo-xin He

**Affiliations:** 1 The Third Affiliated Hospital of Wenzhou Medical University, Wenzhou, China; 2 The First Affiliated Hospital of Wenzhou Medical University, Wenzhou, China; 3 Department of Hyperbaric Oxygenation, The Third Affiliated Hospital of Wenzhou Medical University, Wenzhou, China; 4 Department of Radiology, The Third Affiliated Hospital of Wenzhou Medical University, Wenzhou, China; 5 Department of Intensive Care Unit, The Third Affiliated Hospital of Wenzhou Medical University, Wenzhou, China

**Keywords:** brain Injury, follow-up imaging, heart stroke, hyperbaric oxygen therapy, neurological recovery

## Abstract

**Background:**

Heat stroke (HS) is a critical condition involving severe central nervous system injury. While standard treatments—such as cooling, ventilation, and fluid resuscitation—can stabilize patients, they are often inadequate in addressing long-term neurological complications, including impaired consciousness and paralysis. The persistent deficits in neurological function remain a major clinical challenge. Hyperbaric oxygen therapy (HBOT), which improves brain oxygenation and supports neural repair, offers a potential solution. In the case we present of a patient with HS and brain injury, HBOT was associated with neurological recovery, although its specific contribution relative to conventional treatment remains uncertain.

**Case presentation:**

A 35-year-old male laborer was admitted to the hospital following 5 h of outdoor exertion in high ambient temperatures. He presented comatose with a GCS of 3 (E1V1M1), with sluggish pupillary light reflexes and absent Babinski signs. Initial management in the intensive care unit included active cooling, mechanical ventilation, and supportive therapy. Despite these measures, the patient remained unconscious with quadriparesis. Cranial CT and MRI indicated signal abnormalities in the bilateral cerebellar hemispheres and basal ganglia, which were consistent with HS-induced encephalopathy. Upon stabilization of core temperature, the patient started HBOT at 0.20 MPa for 60–80 min daily in 10-day cycles. After 27 sessions, the patient exhibited progressive neurological improvement, including recovery of consciousness, restoration of limb strength, assisted ambulation, and partial return of speech. The patient was ultimately discharged in accordance with medical advice.

**Conclusions:**

In this case of classic heatstroke with cerebral involvement, HBOT was introduced after partial neurological improvement had already occurred, and was associated with further recovery. The precise role of HBOT in addition to supportive care remains to be clarified.

## 1 Background

Heat stroke (HS) is a life-threatening condition characterized by a core body temperature exceeding 40°C, as well as central nervous system (CNS) dysfunction, including delirium, seizures, or coma (1). Heatstroke is classified as classic when it results from prolonged passive exposure to high environmental temperatures; and heatstroke is classified as exertional when it is triggered by intense physical activity (2). In this case, the features were more consistent with classic heatstroke, as the patient worked in a hot environment with dry skin. Classic heatstroke occurs in epidemic form and contributes to 9–37% of heat-related fatalities during heatwaves. Classic heatstroke mortality can reach 63.2% despite intensive care (3). Due to ongoing effects of global warming, abnormal temperature extremes, and inadequate power infrastructure, heatstroke incidence has increased, and cases involving multi-organ dysfunction have risen (4).

Interestingly, heatstroke’s clinical manifestations are diverse and can involve damage to multiple systems. Notably, CNS injury is a common manifestation of heatstroke (5), it is one of the diagnostic criteria of HS and has been reported frequently, including in large reviews (6). However, its imaging and clinical spectrum are variable, which can complicate diagnosis and management. Hyperbaric oxygen therapy (HBOT) can provide oxygen supply to damaged tissues, promote angiogenesis and tissue repair, reduce inflammation and apoptosis, and improve neurogenesis and cerebral blood flow (7–9). HBOT involves inhaling pure oxygen or high-concentration oxygen in an environment with higher-than-normal atmospheric pressure (10). Moreover, HBOT has been reported to promote neurological function recovery for brain injury caused by heatstroke (11). However, clinical reports on HBOT are rare, and its therapeutic efficacy is unclear. In the case we present here, a patient with severe classic heatstroke complicated by brain injury achieved improvement after HBOT. This case may provide insights into the diagnosis and management of HS-induced brain dysfunction.

## 2 Case presentation

A 35-year-old male laborer was admitted following 5 h of unconsciousness after prolonged outdoor work in the extreme heat. On July 17, 2022, he developed hyperthermia and impaired consciousness while working outdoors and was transported to a nearby tertiary hospital for emergency care. On admission, he was comatose with a GCS of 3 (E1V1M1). The patient’s vital signs included the following: temperature 41.9°C, heart rate 98 bpm, respiratory rate 40 breaths/min, and blood pressure 71/35 mmHg. The patient’s pupils were bilaterally 3.0 mm with sluggish light reflexes. Upon pulmonary examination, we identified coarse breath sounds without rales; and his cardiac examination was unremarkable. Although the patient’s muscle tone was normal, his muscle strength could not be assessed. The skin was dry without petechiae, and Babinski signs were negative bilaterally. In addition, his electrocardiography showed ST-segment changes; though his initial cranial CT was unremarkable. The patient’s chest CT indicated bilateral patchy pulmonary infiltrates and pleural thickening with effusion. The patient was diagnosed with heatstroke, and admitted to the intensive care unit on the same day. During treatment, core body temperature was reduced from 41.9 to 38.5°C within approximately 4 h using physical cooling methods, including ice blankets, ice caps, ice-cold intravenous saline infusion, and targeted ice application over major arteries, while supportive care measures included fluid resuscitation, endotracheal intubation, infection control, management of cerebral edema, and protection of hepatic and renal function. Table 1 shows the serial investigation results during admission, which depict the affected organ systems.

**TABLE 1:** Serial investigation results.

Parameters	Reference range	On admission (day 1)	Before the initiation of HBOT (day 17)	Before discharge
SpO2	91.9–99.0	91.7	98.6	98.0
White blood cell (× 10^9^/L)	3.5–9.5	5.0	6.1	3.9
Hemoglobin (g/L)	130–175	96	73	96
Platelet count (× 10^9^/L)	125–350	128	445	243
AST (U/L)	9–50	96	91	25
ALT (U/L)	15–40	139	74	19
Creatinine (μmol/L)	57–97	169	192.2	49
CRP (mg/L)	0.00–8.00	0.61	42.6	3.7
Lactate (mmol/L)	0.5–1.7	7.7	1.2	\
PCT	0.00–0.50	5.92	\	\
APTT	10.0–13.5	12.5	11.9	\
LDH	120–250	1,643	381	\

ALT, alanine aminotransferase; AST, aspartate aminotransferase; CRP, C-reactive protein; PCT, Procalcitonin; APTT, activated partial thromboplastin time; LDH, Lactate Dehydrogenase.

**FIGURE 1:**
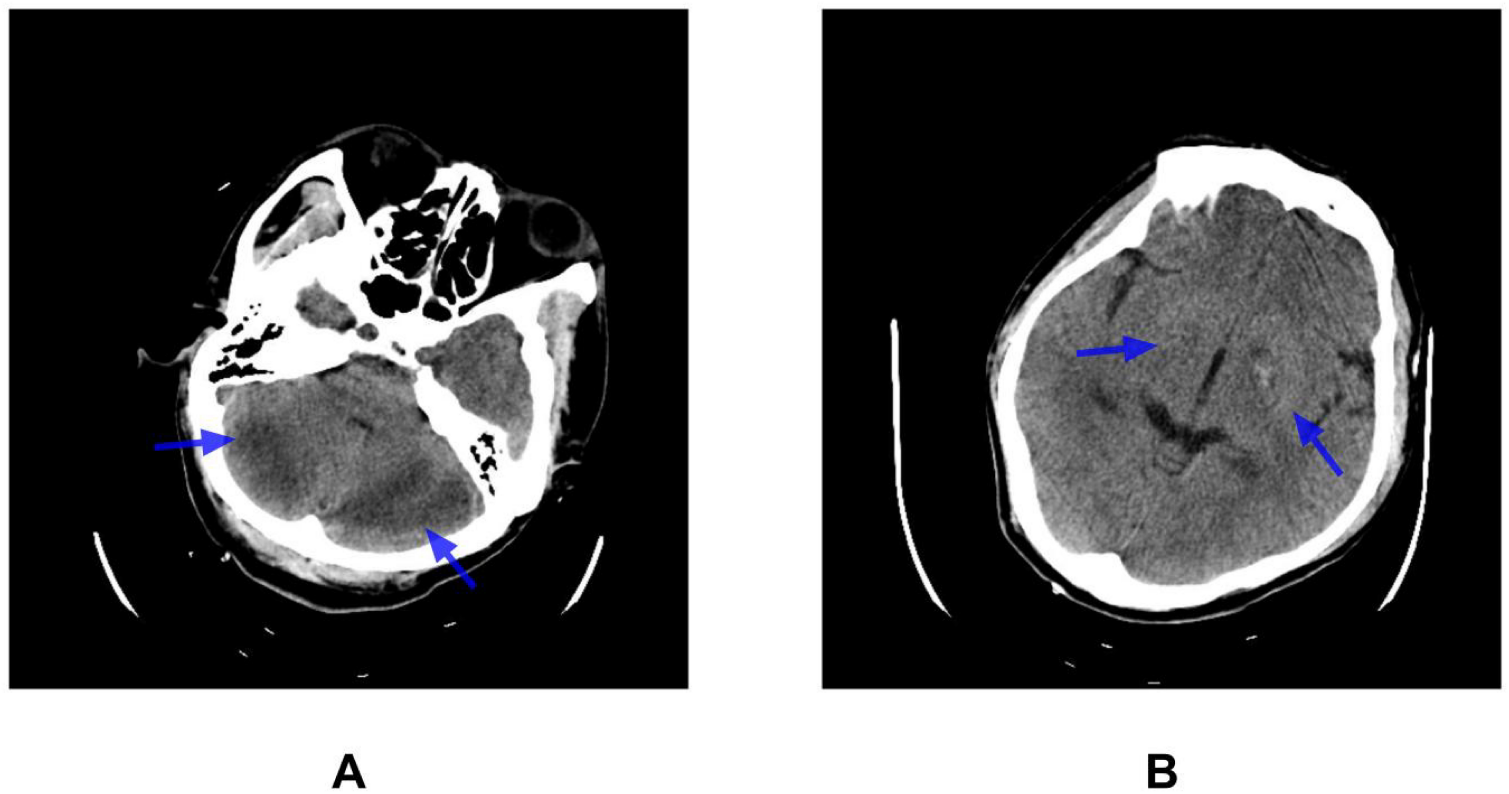
Brain CT findings (day 3). CT scan showing **(A)** bilateral hypodense lesions in the cerebellar hemispheres and **(B)** mixed-density lesions in the bilateral basal ganglia, as indicated by the blue arrows.

**FIGURE 2:**
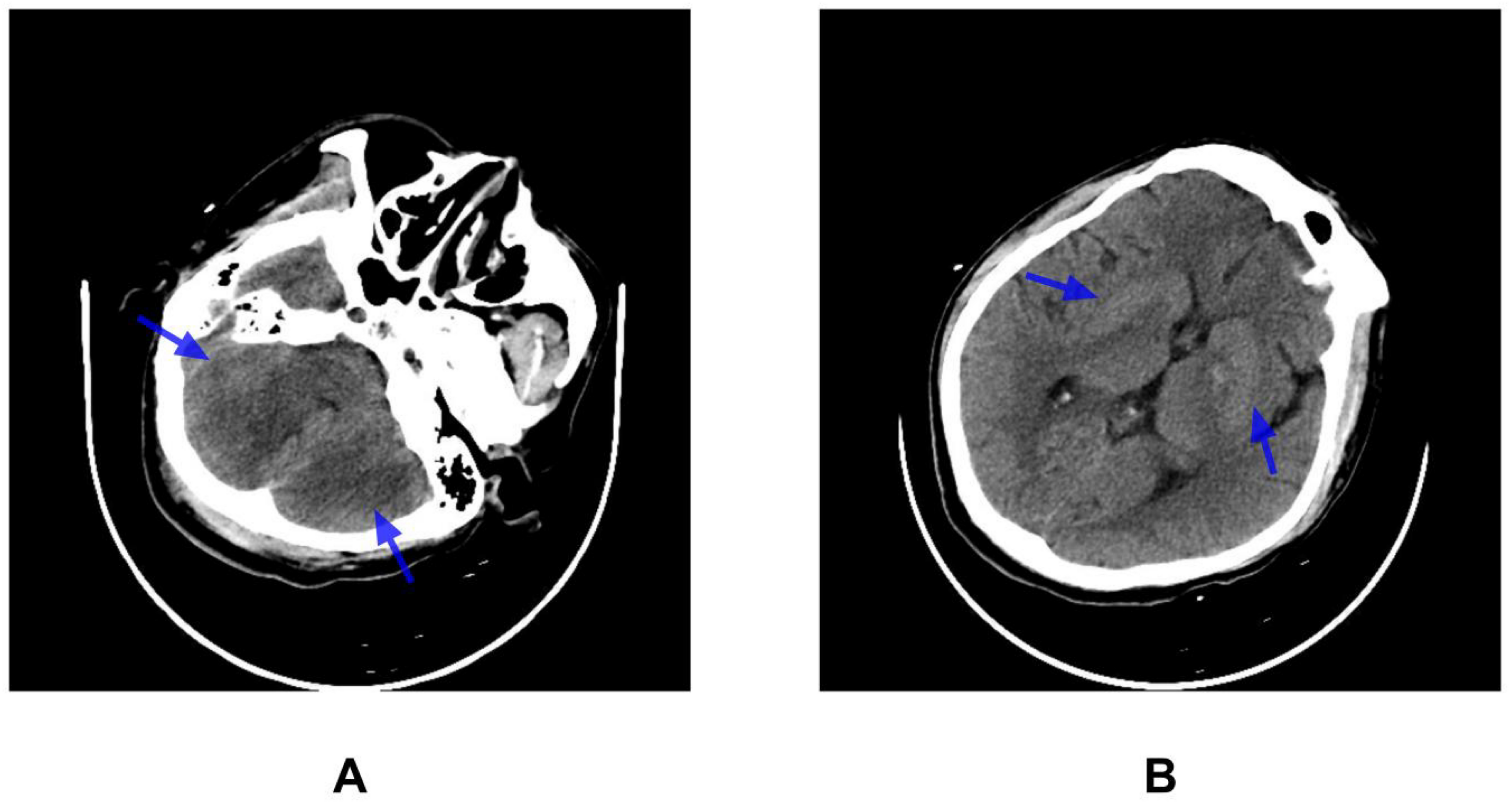
Brain CT findings (day 9). CT scan showing **(A)** bilateral hypodense lesions in the cerebellar hemispheres and **(B)** slightly hyperdense mixed-density lesions in the bilateral basal ganglia, as indicated by the blue arrows. These findings are largely unchanged compared with the CT performed on July 19, 2022.

Despite active cooling and mechanical ventilation, the patient remained comatose with a GCS of 4 (E1V1M2) and persistent hyperthermia (38.5–39.2°C). Pupillary reflexes were preserved, the neck was supple, and no pathological reflexes were elicited. The patient exhibited no voluntary movement in the upper and lower limbs, but pain-induced muscle contractions were observed without specific localization. Muscle strength was graded as 1/5 in the upper limbs and 1/5 in the lower limbs. Repeat CT on hospital day 3 (Figure 1) indicated symmetric low-density lesions in the cerebellum and mixed-density areas in the bilateral basal ganglia. This imaging was consistent with right-sided lacunar infarction. A follow-up CT performed 9 days later (Figure 2) demonstrated no significant interval changes compared with prior CT.

**FIGURE 3:**
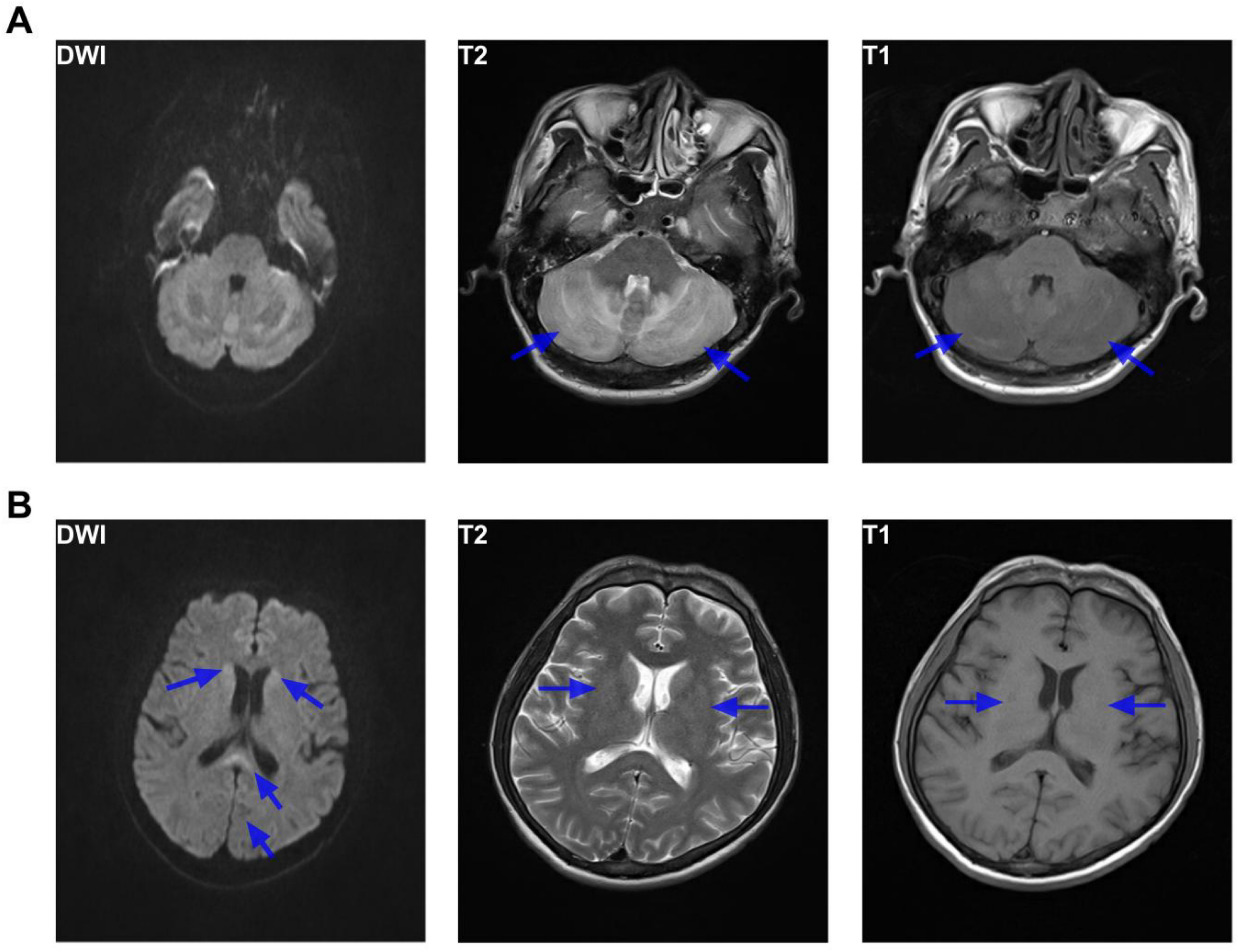
Brain MRI findings (day 12). Brain MRI showing **(A)** patchy mixed T1- and T2-weighted signal abnormalities in the bilateral cerebellar hemispheres, and **(B)** patchy, slightly hyperintense T2-weighted signals in the bilateral basal ganglia. DWI demonstrates small foci of restricted diffusion in the splenium of the corpus callosum, bilateral caudate heads, and left temporo-occipital watershed region, as indicated by the blue arrows.

By July 28, 2022, the patient exhibited neurological improvement with a GCS of 10 (E4V1M5), and was able to open his eyes spontaneously, showing eye-tracking in response to verbal stimuli and localization to pain. However, he remained non-verbal. Facial asymmetry was noted with left nasolabial flattening. His muscle strength was graded as 2/5 in both upper limbs and the left lower limb and 1/5 in the right lower limb. MRI on hospital day 12 (Figure 3) showed patchy T1/T2 signal alterations in both cerebellar hemispheres and symmetrical hyperintensities in the bilateral basal ganglia. In addition, diffusion-weighted imaging (DWI) showed restricted diffusion in the splenium of the corpus callosum, bilateral caudate heads, and left temporo-parieto-occipital watershed area.

After the patient’s body temperature had remained stable at approximately 37–38°C for more than 48 h, HBOT was initiated on 3 August 2022 using a multiplace hyperbaric chamber system (YC3200 J-X, Yantai Haote Oxygen Industry Equipment Co., Ltd., Yantai, China). The system consisted of 30 main compartments and 4 transfer compartments. Sessions were conducted at 0.20 MPa with continuous oxygen inhalation, including 60–80 min of oxygen breathing under pressure and a total session duration of approximately 2 h. Treatment was administered once daily, with 10 sessions forming one course, followed by a scheduled 2-day interval before initiation of the subsequent course. A total of 2–4 courses was planned. After 13 sessions of HBOT, the patient’s consciousness gradually improved, limb spasms resolved, and motor function improved, with a GCS of 11 (E4V1M6). Partial active resistance was observed in both upper limbs and the left lower limb, while movement was noted in the right lower limb. Muscle strength was graded as 4/5 in the upper limbs, 4/5 in the left lower limb, and 2/5 in the right lower limb.

**FIGURE 4:**
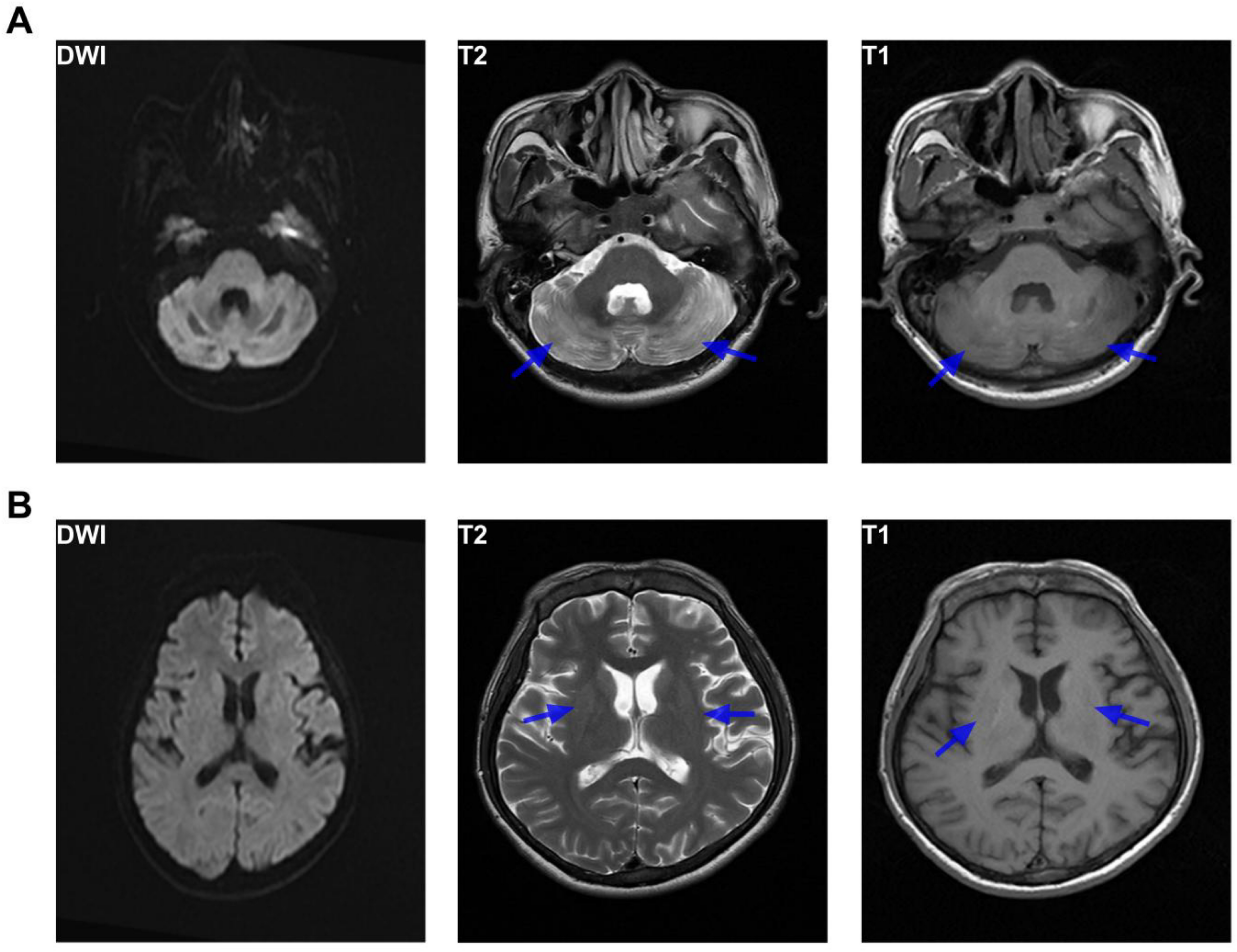
Brain MRI findings (day 51). Brain MRI showing **(A)** small patchy mixed T1- and T2-weighted signal abnormalities in the bilateral cerebellar hemispheres, with internal foci of short T1 signal, and **(B)** patchy mixed T1- and T2-weighted signal abnormalities in the bilateral basal ganglia with symmetrical distribution, as indicated by the blue arrows. DWI shows no obvious areas of high signal. Ventricular, cisternal, and sulcal morphology and positions are preserved, and midline structures are intact. The findings are consistent with typical imaging features of heatstroke. Compared with the MRI performed on 27 July 2022, the lesions show partial resolution and reduction in size.

**FIGURE 5:**
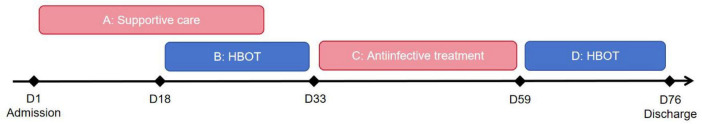
The treatment course of heatstroke complicated by brain injury. **(A)** Supportive care: Cooling measures (ice blanket), fluid infusions, oxygen therapy, anticoagulant therapy, liver and kidney support, prophylactic antibiotics, etc. **(B)** First HBOT course (days 18–33): A total of 13 HBOT sessions were administered before treatment was terminated due to persistent high fever. **(C)** Antiinfective treatment and stabilization (days 33–59): HBOT paused; patient received targeted antimicrobial therapy and continued supportive care. **(D)** Second HBOT course (days 59–76): A total of 14 HBOT sessions were completed during this 18-day course. HBOT was administered once daily for 10 consecutive days per course, with a 2-day interval between consecutive courses, as detailed in the main text.

On August 18, the patient developed a fever, prompting temporary HBOT discontinuation and empiric antimicrobial therapy initiation. By 5 September 2022, follow-up MRI (Figure 4) demonstrated partial resolution of previously noted cerebral lesions, with decreased size and signal intensity. After the patient’s body temperature had remained stable within the normal range for 3 days, HBOT was resumed on September 13. Following 27 HBOT sessions, the patient demonstrated substantial functional recovery. Muscle strength improved to near-normal levels, graded as 5/5 in both upper limbs, 5/5 in the left lower limb, and 4 + /5 in the right lower limb. He was able to ambulate with assistance and could engage in simple verbal communication. The patient was discharged in stable condition on 30 September 2022 (Figure 5 summarizes the treatment course of heatstroke complicated by brain injury).

## 3 Discussion

### 3.1 HS-induced brain injury

HS is a life-threatening condition that can result in multiple organ dysfunction, with the CNS being particularly vulnerable to hyperthermia (12). Current evidence suggests that heatstroke-related brain injury involves cerebral hypoperfusion, oxidative stress, neuroinflammation, and cell death pathways—including apoptosis, autophagy, and ferroptosis—leading to neuronal loss and potential long-term neurological deficits (13). Neurological manifestations commonly include delirium, seizures, and coma, and in severe cases, persistent neurological deficits may ensue (14).

Previous studies have demonstrated that typical neuroimaging findings in HS comprise diffuse cortical edema, cerebellar lesions, and basal ganglia abnormalities—features that are often associated with unfavorable functional outcomes. Furthermore, cerebellar atrophy, basal ganglia damage, and persistent motor or cognitive impairments are frequently reported as long-term sequelae, and the overall prognosis of severe HS-related brain injury remains poor. CNS involvement usually develops within several hours to 1–2 days after HS onset, and abnormal signals on computed tomography (CT) or magnetic resonance imaging (MRI) can sometimes be detected as early as 24–48 h post-onset (15, 16). Delayed complications, including cerebellar atrophy, basal ganglia injury, and progressive motor or cognitive dysfunction, typically emerge gradually over the following several weeks or even later (17).

### 3.2 Treatment of HS-associated brain injury

Cases of heatstroke leading to neurological sequelae, coma, or death remain common, yet no standardized therapy for heatstroke-related brain injury exists. Current management remains centered on prompt and effective cooling, supplemented by fluid resuscitation, organ support measures, infection control, and other targeted supportive treatments (17). Previous case reports have described an elderly female patient with severe non-exertional heatstroke who was initially comatose (GCS = 3). With supportive care, her consciousness gradually recovered, and she was discharged on day 11 with a GCS of 15 and without any neurological sequelae (18). In contrast, in the present case, despite 18 days of conventional management during which vital signs stabilized and other organ functions progressively improved, neurological improvement and imaging changes remained minimal.

Given the limited neurological recovery observed under conventional management, alternative therapies that may enhance brain function are of particular interest.

HBOT is a non-invasive alternative. Early, standardized HBOT can rapidly correct cerebral hypoxia-ischemia, improve consciousness, and enhance neurological outcomes, while also benefiting other organ systems (11). Moreover, HBOT may confer sustained benefits, such as enhanced cognitive recovery and improved overall quality of life (19). Retrospective analyses suggest that HBOT not only significantly reduces mortality compared with conventional therapy (0% vs. 8.49%) in patients with heatstroke, but also improves neurological function, as indicated by GCS and NIHSS scores (11). To contextualize the regimen used in this case, clinical studies conducted in the United States and Europe generally apply HBOT treatment pressures of 0.15–0.25 MPa (1.5–2.5 ATA), with individual sessions lasting 60–140 min and total treatment courses ranging from 20 to 120 sessions (20–23). HBOT protocols for traumatic brain injury (TBI), though not fully standardized, commonly adopt similar pressures delivered once daily in 5–10-session cycles, with total course lengths of 30–60 sessions (24).

In this case, a personalized HBOT regimen, tailored to the patient’s age, clinical severity, and the pressure-duration parameters recommended in current TBI literature, was implemented. Following the initiation of HBOT, the patient’s neurological function and cerebral imaging showed progressive improvement. At discharge, he exhibited restored consciousness, motor, and language functions, and was able to ambulate with assistance. These findings suggest that early, individualized HBOT may facilitate recovery in heatstroke-associated brain injury, although delayed benefits from prior supportive treatments cannot be completely excluded. Further large-scale clinical studies are warranted to validate its efficacy and safety.

### 3.3 Mechanisms HBOT for HS-induced brain injury

HBOT was initially developed from hyperbaric research and has since evolved into a well-established, multidisciplinary therapeutic modality. It is currently applied in conditions such as acute ischemic stroke, neurodegenerative diseases, trauma repair (25), carbon monoxide poisoning (26), and multiple organ dysfunction associated with HS (11). Despite its recognized therapeutic potential across these fields, the use of HBOT in neurological disorders, including HS-related brain injury, remains insufficiently investigated and warrants further clinical validation (27).

Patients in the acute phase of HS often present with high fever and pronounced inflammatory responses; immediate administration of HBOT during this stage may aggravate oxidative stress and thereby exacerbate cellular injury (11). These findings suggest that appropriate timing of HBOT initiation is crucial to maximize its therapeutic efficacy and ensure patient safety.

The neuroprotective effects of HBOT in HS-related brain injury are supported by several mechanistic pathways. These include elevation of brain tissue oxygen tension (PO_2_) which enhances aerobic metabolism and mitigate cytotoxic edema (28); improvement of cerebral perfusion and microcirculatory function to alleviate ischemia (29); and attenuation of inflammatory responses by inhibiting the activation of nuclear factor-κB (NF-κB) and the NOD-like receptor pyrin domain-containing 3 (NLRP3) inflammasome, thereby reducing proinflammatory cytokine levels (30). HBOT may promote neuroregeneration and angiogenesis, and support enhance antioxidant defenses through activation of the nuclear factor erythroid 2-related factor 2 (Nrf2) pathway (31, 32). It may also help maintain hypothalamic thermoregulation and systemic homeostasis, collectively contributing to neurological recovery (33).

In this case, the patient’s neurological recovery likely reflects multiple beneficial effects of HBOT. Improved consciousness may result from enhanced cerebral oxygenation and restored neuronal metabolism, while the gradual recovery of limb strength and resolution of spasms suggest improved perfusion and reduced neuroinflammation. The partial resolution of MRI lesions corresponds with HBOT-induced neuroregeneration, angiogenesis, and antioxidant activity. These observations highlight HBOT’s potential to promote comprehensive neural recovery in heatstroke-related brain injury.

## 4 Limitation

Although peripheral nerve or muscle injury may also contribute to motor dysfunction after HS (34, 35), this possibility cannot be fully excluded. However, the pattern of neurological deficits, neuroimaging findings, and the patient’s favorable response to HBOT were more indicative of CNS involvement.

## 5 Conclusion

In conclusion, this case underscores the potential reversibility of severe HS-related brain injury and the therapeutic benefits of HBOT in combination with conventional treatment. It also highlights the value of long-term clinical and neuroimaging follow-up to monitor recovery trajectories and guide ongoing management. Although preliminary findings are promising, well-designed clinical trials are required to further validate the efficacy of HBOT, define the optimal initiation window, pressure-duration parameters, and treatment course, and clarify its long-term safety and effectiveness in HS-related brain injury.

## Data Availability

The original contributions presented in this study are included in this article/supplementary material, further inquiries can be directed to the corresponding author.
